# The heel fat pad: mechanical properties and clinical applications

**DOI:** 10.1186/1757-1146-4-S1-I14

**Published:** 2011-05-20

**Authors:** Scott C Wearing, James E Smeathers

**Affiliations:** 1 Faculty of Health Sciences and Medicine, Bond University, Gold Coast, Qld, 4229, Australia; 2 Centre of Excellence for Applied Sports Science Research, Queensland Academy Sport, Nathan, Qld, 4111, Australia; 3 Institute of Health and Biomedical Innovation, Queensland University of Technology, Brisbane, Qld, 4059, Australia

## 

The human heel pad is a highly specialised fibroadipose tissue that is hierarchically structured to dissipate the stress associated with weight–bearing activities. While the properties of the heel pad, as a whole, are believed to reflect those of the collagen– and elastin–rich septa which envelope adipocytes and confine their movement, the scientific literature provides little consensus on the properties of healthy heel pads. Experiments conducted *in vitro* typically yield stiffness and loss properties that differ by an order of magnitude to those performed *in vivo*. Such differences may, in part, reflect the difficulty in measuring heel pad mechanics *in vivo*. This paper reports the findings of a novel series of experiments in which a digital fluoroscope, synchronised with a pressure platform, was used to obtain force–deformation data of the heel pad during gait. Transient loading profiles associated with walking were observed to induce rapidly changing deformation rates in the heel pad and resulted in irregular load–deformation curves. Initial stiffness (32 N.mm^-1^) of the heel pad was an order of magnitude lower than its final stiffness (212 N.mm^-1^), which, in turn, was similar to that reported for cadaveric heel pads (296 N.mm^-1^) when impacted at comparable energies of 1.45 J. While the energy dissipating ratio of the heel pad (0.66 ± 0.12) fell between those commonly cited for mechanical tests of cadaveric heels and impact loading *in vivo*, peak deformation of the fat pad (10.3 mm) approached that predicted for the limit of pain tolerance (10.7 mm), suggesting that the heel pad operates near its physiological maximum, even at the relatively modest speeds encountered during walking. In plantar heel pain, the elastic properties of the heel pad remained unaltered. However, energy loss within the tissue was reduced in symptomatic limbs and was also correlated with the sonographic thickness of the plantar fascial enthesis. These findings suggest that viscosity, rather than elasticity, of the heel fat pad may play an important role in the severity of heel pain and provides a previously unidentified link between the mechanical behaviour of the plantar fat pad and plantar heel pain.

